# Osteopontin is An Important Regulative Component of the Fetal Bone Marrow Hematopoietic Stem Cell Niche

**DOI:** 10.3390/cells8090985

**Published:** 2019-08-27

**Authors:** Huimin Cao, Benjamin Cao, Chad K. Heazlewood, Melanie Domingues, Xuan Sun, Emmanuel Debele, Narelle E. McGregor, Natalie A. Sims, Shen Y. Heazlewood, Susan K. Nilsson

**Affiliations:** 1 Biomedical Manufacturing Commonwealth Scientific and Industrial Research Organisation (CSIRO), Clayton, VIC 3800, Australia; 2 Australian Regenerative Medicine Institute, Monash University, Clayton, VIC 3800, Australia; 3 St. Vincent’s Institute of Medical Research, Fitzroy, VIC 3065, Australia; 4 The University of Melbourne, Department of Medicine at St. Vincent’s Hospital, Fitzroy, VIC 3065, Australia

**Keywords:** osteopontin, fetal, hematopoietic stem cells, secreted phosphoprotein 1

## Abstract

Osteopontin (OPN) is an important component in both bone and blood regulation, functioning as a bridge between the two. Previously, thrombin-cleaved osteopontin (trOPN), the dominant form of OPN in adult bone marrow (BM), was demonstrated to be a critical negative regulator of adult hematopoietic stem cells (HSC) via interactions with α_4_β_1_ and α_9_β_1_ integrins. We now demonstrate OPN is also required for fetal hematopoiesis in maintaining the HSC and progenitor pool in fetal BM. Specifically, we showed that trOPN is highly expressed in fetal BM and its receptors, α_4_β_1_ and α_9_β_1_ integrins, are both highly expressed and endogenously activated on fetal BM HSC and progenitors. Notably, the endogenous activation of integrins expressed by HSC was attributed to high concentrations of three divalent metal cations, Ca^2+^, Mg^2+^ and Mn^2+^, which were highly prevalent in developing fetal BM. In contrast, minimal levels of OPN were detected in fetal liver, and α_4_β_1_ and α_9_β_1_ integrins expressed by fetal liver HSC were not in the activated state, thereby permitting the massive expansion of HSC and progenitors required during early fetal hematopoiesis. Consistent with these results, no differences in the number or composition of hematopoietic cells in the liver of fetal OPN^-/-^ mice were detected, but significant increases in the hematopoietic progenitor pool in fetal BM as well as an increase in the BM HSC pool following birth and into adulthood were observed. Together, the data demonstrates OPN is a necessary negative regulator of fetal and neonatal BM progenitors and HSC, and it exhibits preserved regulatory roles during early development, adulthood and ageing.

## 1. Introduction

Osteopontin (OPN) is a phosphorylated glycoprotein encoded by a single gene in a cluster of “SIBLING” (small integrin-binding ligands) family proteins, expressed by a broad range of cells in different tissues. Nascent OPN is ~33 KDa in size and is comprised of ~300 amino acids (297 in mouse; 314 in human); it is post-translationally modified [1] via phosphorylation, glycosylation and proteolytic cleavage [2]. Also known as secreted phosphoprotein 1 (*SPP1*), OPN is a cytokine involved in many physiological and pathological processes including cell adhesion [3], angiogenesis [4], apoptosis [5], chemotaxis [6,7] and tumor metastasis [8]. 

In adult bone marrow (BM), bone is the major source of OPN. It is secreted by bone surface cells, including osteoblasts during bone formation [9,10], and osteoclasts during bone resorption [11], and by osteocytes, which reside within the bone matrix [9,10]. OPN is also synthesized by osteoblast precursors [9,10] as well as hematopoietic cells including osteoclast precursors, macrophages [12] and hematopoietic progenitors [5]. In adult bone, OPN expression is restricted to the bone and BM interface (endosteal region) [5,13,14,15] where it plays pivotal roles in adult hematopoiesis, providing both a physical structure as well as interacting with hematopoietic and non-hematopoietic cellular and extracellular molecules of the HSC niche [16]. Previous studies showed that the transplantation of wild-type (WT) hematopoietic stem and progenitor cells into OPN knockout (OPN^-/-^) mice results in a significant reduction in the number of donor progenitors lodging in the endosteal region, leading to defective engraftment [13]. In addition, OPN^-/-^ mice have a significantly larger BM HSC pool, highlighting OPN as a potent negative regulator of HSC [5,13]. Notably, trOPN, the dominant form of OPN present in human and murine BM, but not full length OPN, binds to α_4_β_1_ and α_9_β_1_ integrins expressed by HSC [17] and leads to the inhibition of progenitor proliferation and differentiation in vitro [13]. Furthermore, the absence of OPN alters the expression of other growth factors, cytokines and surface molecules known to regulate HSC, including stromal cell derived factor-1 alpha (SDF-1α) [18], vascular endothelial growth factor (VEGF) [19], jagged-1 and angiopoietin [5] and modifies cell sensitivity to cytokine stimuli such as interleukin 3 and granulocyte-macrophage colony-stimulating factor [20], both validating OPN as a critical regulator of HSC via direct and indirect mechanisms. More recently, megakaryocytes in adult BM were identified as the major local source of Factor V (FV), factor X (FX) and prothrombin (PT), which are required for the production of trOPN [21].

OPN is also expressed in sites of hematopoiesis during embryogenesis [22,23], although its specific role in HSC regulation in early development is unknown. To establish the adult HSC pool, developing fetal HSC initially expand in the fetal liver, then colonize the fetal BM, prior to the HSC pool becoming quiescent, to maintain homeostasis throughout adulthood; at the individual HSC level, cells cycle continuously, albeit very slowly [24]. Like adult BM, each individual fetal hematopoietic microenvironment possesses unique and critical factors which educate resident HSC [25]. During ontogeny, the primary site for fetal hematopoiesis is the liver and although fetal liver stromal cells have been shown to express OPN in culture, it is not known whether fetal liver OPN is important in the regulation and migration of HSC in vivo [23]. Furthermore, as in the adult, a major source of OPN in fetal BM is osteoblast lineage cells—exemplified by a significantly decreased expression of OPN on BM cells from osterix null mice and failure of these cells to support long-term HSC [26]. OPN is also a pivotal component in vasculogenesis [19,27], a key process in fetal bone development which indirectly regulates fetal hematopoiesis. The role of OPN in fetal HSC regulation during early development is poorly understood. 

In the current study, we demonstrate a critical role for OPN in the regulation of hematopoietic stem and progenitor cells in fetal and neonatal BM. Notably, we demonstrate OPN, and specifically trOPN, is expressed in fetal BM but not fetal liver, and the trOPN receptors, α_4_β_1_ and α_9_β_1_ integrins, are highly expressed by fetal HSC. These integrins were endogenously activated by the presence of divalent metal cations in the BM microenvironment; a characteristic not observed in fetal liver. Similar to adult BM, OPN is a negative regulator of neonatal HSC proliferation, maintaining the fetal BM stem and progenitor cell pool. Together, our results demonstrate that the absence of OPN and the reduced activity of α_4_β_1_ and α_9_β_1_ integrins on HSC in fetal liver permits the massive expansion of stem and progenitors required during early fetal hematopoiesis. In contrast, the large amounts of OPN/trOPN as well as integrin-activating cations present in fetal BM constitute a microenvironment that facilitates HSC migration into the marrow and provides the necessary signals to mediate HSC quiescence for maintenance of the stem cell pool after birth and throughout adulthood. 

## 2. Methods

### 2.1. Mice

OPN^-/-^ mice devoid of all OPN isoforms (*Spp1*^tm1Blh^) [28] and wild-type controls were bred on a C57BL/6J (C57, Ly5.2) background at the Monash Animal Research Platform (Monash University, Melbourne, Australia). All experiments were approved by the Monash Animal Research Platform ethics committee. Mice were harvested from embryonic (E) 14.5 until newborn day (D) 4, with timed pregnancies set up as previously described [29].

### 2.2. Immunohistochemistry

Isolated fetal femurs were immersion-fixed in 4% paraformaldehyde for 24 hours at room temperature without vacuum before being transferred to Sorensen’s buffer (0.1 M with 5% sucrose). Tissues were processed into paraffin wax and staining was performed as previously described [21]. Anti-OPN (R&D Systems, Minneapolis, USA; goat IgG), factor X (FX)and prothrombin (PT) (in-house biotinylated; Quanta Biodesign, Plain City, USA; sheep IgG) were used at 1, 2 and 0.5 µg/mL, respectively. Staining for FX and PT was followed by amplification using a PerkinElmer TSA kit (Waltham, USA). 

### 2.3. Cell Lysis and OPN/trOPN Quantification via ELISA

Fetal organs were dissociated in PBS and separated into supernatant and pellet fractions by centrifugation. The supernatant was assessed for the presence of soluble proteins while the pellet was lysed as previously described and assessed for cell membrane-bound/intracellular proteins and non-soluble proteins [29]. OPN and trOPN expression were assayed in duplicates from three biological repeats using either an OPN (R&D) or trOPN (IBL, Fujioka-Shi, Japan) ELISA kit according to the manufacturer’s instructions. 

### 2.4. Fetal Liver HSC In vitro Proliferation Analysis

Fetal liver from either E14.5 C57 or OPN^-/-^ pups were harvested, passed through a 40 μm strainer to harvest single cells and low density (<1.077 g/cm^3^) mononuclear cells were then obtained by discontinuous density centrifugation. The cells were depleted for B220^+^ and Gr1^+^ cells through negative selection using rat-anti-mouse B220 and Gr1 (BD Pharmingen, Franklin Lakes, USA) antibodies followed by sheep anti-rat immunoglobulin G (IgG) Dynabeads (Invitrogen, Carlsbad, USA). The depleted cells were stained with an antibody cocktail containing lineage antibodies (anti-CD3, B220, Gr1, TER119; all APCCy7 conjugated), anti-ckit-AF647, Sca-1-PECy7, CD150-PE and CD48-FITC and 75 lineage^neg^Sca-1^+^ckit^+^CD150^+^CD48^−^ (LSKSLAM) cells were sorted directly into a 96-well plate containing serum free Iscove’s Modified Dulbecco’s Medium (IMDM; Invitrogen) supplemented with 1% BSA, insulin (10 µg/mL; Novartis, Basel, Switzerland), Transferrin (100 µg/mL; Roche, Basel, Switzerland), low-density lipoprotein (LDL) (3 µg/mL; Organon Teknika-Cappel, Malvern, UK), L-Glutamine (2 mmol/L; Invitrogen), recombinant mouse stem cell factor (rm-SCF, 10 ng/mL; Millipore, Burlington, USA), recombinant mouse Interleukin-3 (rmIL-3; 133 U/mL), recombinant mouse Interleukin-11 (rmIL-11; 100 ng/mL), human TPO (5 ng/mL), recombinant human IL-6 (rhIL-6; 10 ng/mL), recombinant human Flt-3 (rhFlt-3; 10 ng/mL) (all purchased from PeproTech, Rehovot, Israel) with or without trOPN (15 µg/mL). The cells were cultured for 6 days at 37 °C (5% O_2_, 10% CO_2_ in 85% N_2_) and then progeny cells were harvested and enumerated using a haemocytometer.

### 2.5. Fetal Liver HSC Homing Analysis

Fetal liver HSC (LSKSLAM) were sort purified from E17.5 C57 pups as described above and then stained with the tracking dye seminaphtorhodafluor-1-carboxylic acid acetate succinimidyl ester (SNARF) [30]. The labelled fetal liver HSC (approximately 2000–6500 LSKSLAM cells/recipient) were combined with adult whole BM filler cells (2 × 10^5^/recipient) in PBS and injected into either WT or OPN^-/-^ D2 neonatal pups via intraperitoneal injection (10ul/recipient). After 15 hours, recipients were culled and BM were dissected, processed to single cell and analysed for SNARF events by flow cytometry. Homing efficiency into BM was calculated as the number of donor HSC that homed to the BM divided by the number of donor HSC transplanted. 

### 2.6. Cell Cycle Analysis on Fetal HSC and Progenitors

Fetal progenitors and HSC were analyzed as previously described [31]. In a subset of experiments, HSC and progenitors were further analyzed for cell cycle by staining with Hoechst (Invitrogen) and Ki67 (Pharmingen) using FIX&PERM cell permeabilization reagents (Invitrogen). Specifically, 2 × 10^6^ cells were sequentially incubated in 50 µL fixation medium (A) for 15 min then 50 µL permeabilization medium (B) with Hoechst (20 µg/mL) and Ki67 (5 µL/test) for 30 min at room temperature. Cells stained with Hoechst alone were used as a negative control for Ki67. 

### 2.7. Expression of α_4_β_1_ and α_9_β_1_ on Fetal HSC and Progenitors

For analysis of α_4_β_1_ and α_9_β_1_ integrin expression, HSC and progenitors were stained with anti-α_4_ (CD49d-biotin, rat IgG2a, 10 µg/mL; Pharmingen), anti-β_1_ (CD29-purified, goat IgG, 10 µg/mL; R&D) or anti-α_9_ (α_9_-PE, goat IgG, 0.5 µg/mL; R&D) antibodies, followed by streptavidin BUV805 or anti-goat AF488 secondary antibody as appropriate. Isotype controls were performed in parallel. In vitro R-BC154 binding was assessed as previously described [32]. Specifically, cells were stained with R-BC154 (100 nM) alone or in the presence of excess amounts of the selective α_4_β_1_ antagonist Bio5192 (1µM) or dual α_4_β_1_ and α_9_β_1_ antagonist BOP (1 µM) in PBS containing 0.5%BSA with or without Ca^2+^/Mg^2+^ at 0 °C for 30 min. 

### 2.8. Flow Cytometry

Flow cytometric analysis was done using a LSRII and cell sorting used a Cytopeia Influx (BD Biosciences, Sydney, Australia) equipped as previously described [32]. For generic analysis, cells were analysed at 10–20,000 cells per second except for cell cycle analysis which were analysed at 2000 cells per second. Cell sorting was performed at ~20,000 cells per second and re-analysed to confirm purity (>95%).

### 2.9. Quantification of Calcium, Magnesium and Manganese using Inductively Coupled Plasma Mass Spectrometry (ICPMS) 

Isolated fetal and neonatal organs were processed to single cells as previously described [33] followed by centrifugation to obtain the cell pellets. Then the cell pellets were frozen at −80 °C and processed as previously described [32] for the measurement of levels of calcium, magnesium and manganese in fetal organs using ICPMS. Results were calculated as micrograms of metal per gram of wet weight (µg/g) for comparison among different samples. 

### 2.10. Statistical Analysis and Data Presentation

Flow cytometric analysis was performed using FlowJo and statistical analysis using a two-way ANOVA for the comparison between WT and OPN^-/-^ samples among different age groups, a one-way ANOVA for the comparison for WT samples among different age groups (if the difference between the means was statistically significant, multiple comparison was performed using Tukey’s multiple comparisons test), and t-test for the comparison between WT and OPN^-/-^ samples at a single age. For all tests, normal distribution and equal variance are confirmed. *p* < 0.05 was considered significant. 

## 3. Results

### 3.1. OPN and Specifically trOPN is Highly Expressed in Fetal BM

**Figure 1:**
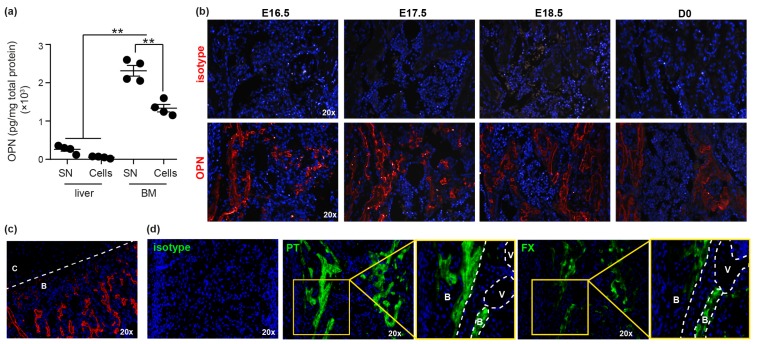
Osteopontin (OPN) is highly expressed in fetal BM. (**a**) OPN protein in E17.5 fetal liver and bone marrow (BM) was quantified using an OPN ELISA (R&D; MOST00). SN: supernatant. ** *p* < 0.01. (**b**) Immunohistochemical analysis of mouse E16.5, E17.5, E18.5 and D0 BM stained with either isotype control or anti-OPN (red). Grey areas represent autofluorescence. (**c**) E17.5 BM demonstrating lack of OPN expression in growth plate cartilage (C) compared to bone (B). (**d**) Immunohistochemical analysis of mouse E17.5 BM stained with either isotype control or anti-prothrombin (PT) and anti-factor X (FX). White dotted lines delineate the structures of the fetal femurs. B: bone; V: blood vessel; C: cartilage.

Initially, the presence of OPN in fetal tissue was determined by ELISA, demonstrating significantly greater levels of OPN in fetal BM compared to liver (Figure 1a). Notably, negligible OPN was detected in fetal liver, suggesting it does not play a direct role in regulating fetal liver HSC during early development. The anatomical location of OPN in fetal BM was then assessed using immunohistochemical staining (IHC), demonstrating significant OPN deposition on surfaces of trabecular bone (Figure 1b), reflective of expression patterns in the metaphysis of adult bones [17]. In addition, OPN was not detected in fetal growth plate cartilage (Figure 1c), which is consistent with previous reports demonstrating a lack of OPN transcript in chondrocytes [34]. In adult BM, the dominant form of OPN is thrombin-cleaved, which regulates HSC and progenitors through interactions with α_4_β_1_ and α_9_β_1_ integrins [17]. trOPN was present in both the supernatant (0.67 ± 0.1 pmol/mg; n = 3) and cellular fraction of fetal BM (0.90 ± 0.1 pmol/mg; n = 3). Furthermore, PT and FX, important factors involved in the production of active thrombin [21], were also observed in fetal BM, with their localisation predominantly in trabecular bone and at the bone/BM interface (Figure 1d). 

### 3.2. OPN is Important in Maintaining the Fetal BM Progenitor Pool

**Figure 2:**
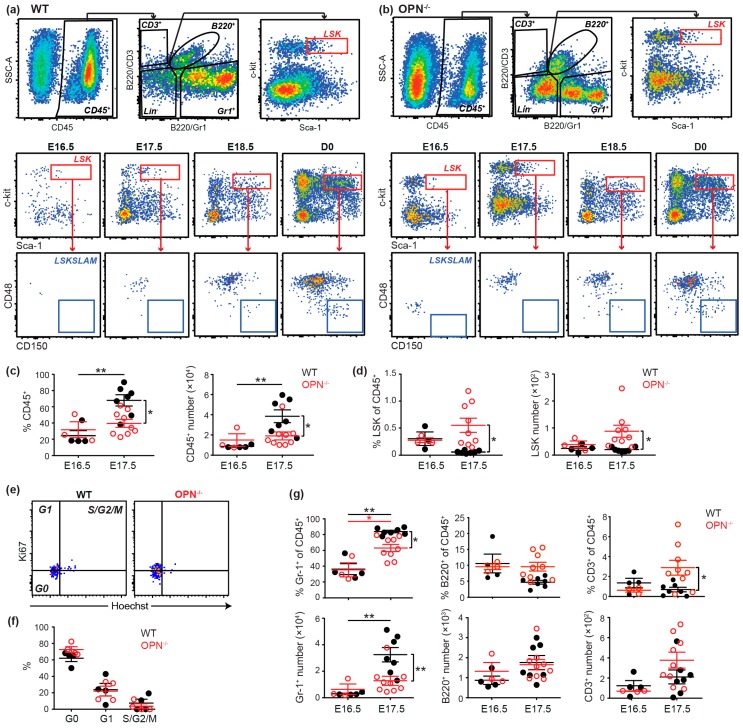
OPN is important in the maintenance of fetal progenitor pools. (**a**) A representative flow cytometric analysis of CD45^+^ hematopoietic cells, lineage positive cells, LSK progenitors (red gate) and SLAM hematopoietic stem cells (HSC) (blue gate) in fetal BM of wild-type (WT) and (**b**) OPN^-/-^ mice. (**c**) Comparison of incidence and content of CD45^+^ and (**d**) LSK progenitors in E16.5 and E17.5 fetal BM of WT and OPN^-/-^ mice. (**e**) Representative cell cycle analysis profile with ki67 and Hoechst on E17.5 BM LSK cells (concatenated n = 2 for WT and n = 3 for OPN^-/-^) and (**f**) incidence of WT and OPN^-/-^ E17.5 fetal BM cells in G0, G1 and S/G2/M. (**g**) Incidence and content of Gr-1^+^ granulocytes, CD3^+^ T-cells and B220^+^ B-cells in E16.5 and E17.5 fetal BM of WT and OPN^-/-^ mice. Data represent the cell content after normalization using mouse weights. Each dot represents the average value for a litter (for fetal) or an individual mouse from different litters (for newborn). WT: closed black circle; OPN^-/-^: open red circle; * *p* < 0.05, ** *p* < 0.01. Data shows mean ± SEM, n ≥ 3.

Consistent with previous findings [35], hematopoietic progenitors (LSK cells) were present in fetal E16.5 BM, but HSC (LSKSLAM cells) were not evident prior to E18.5 (Figure 2a). In the absence of OPN, significantly fewer CD45^+^ hematopoietic cells were evident in the fetal BM at E17.5, but surprisingly this was accompanied by a significantly increased frequency and number of progenitors (Figure 2b–d). Importantly, cell cycle analysis demonstrated this increased progenitor pool was not due to increased proliferation (Figure 2e,f). In contrast, analysis of lineage commitment in the absence of OPN revealed a significant decrease in the incidence and number of granulocytes (Gr1^+^ cells) in fetal BM at E17.5 (Figure 2g), but no differences in the number of B- or T- lymphocytes (B220^+^ and CD3^+^, respectively) (Figure 2g), despite an increase in the proportion of CD3^+^ cells. Collectively, the data suggests the enlarged progenitor pool in E17.5 OPN^-/-^ fetal BM is due to a differentiation defect of progenitors to myeloid/granulocytic cells. 

In contrast, in fetal liver, which is the main reservoir of hematopoietic progenitors and HSC during embryogenesis, no significant differences in the incidence or content of hematopoietic cells (CD45^+^), granulocytes (Gr1^+^) or HSC and progenitors (SLAM and LSK cells) were observed between OPN^-/-^ and WT mice (Figure S1a,b). Similarly, no differences in the cell cycle of fetal liver HSC or progenitors were evident (Figure S1c,d). Nevertheless, prospectively isolated fetal liver HSC exhibited significantly reduced proliferation in the presence of trOPN following 6 days of culture (Figure S1e). Together, the data demonstrate fetal liver HSC are still capable of responding to the negative regulatory role of trOPN, but owing to its low expression (Figure 1a), its impact in fetal liver hematopoiesis in situ is limited. 

### 3.3. OPN Regulates the HSC Pool in Neonatal BM.

**Figure 3:**
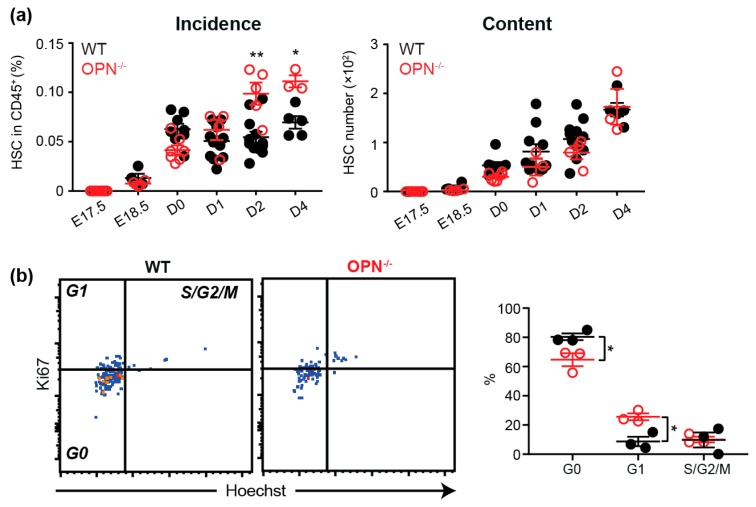
OPN is important in the maintenance of fetal HSC pools. (**a**) A summary of incidence and content of SLAM HSC in the fetal and neonatal BM. Data represents the cell content after normalization by mouse weights. (**b**) Cell cycle analysis with Ki67 and Hoechst on D2 BM LSKSLAM cells. Each dot represents the average value for a litter (for fetal) or an individual mouse from different litters (for newborn). WT: closed black circle; OPN^-/-^: open red circle; * *p* < 0.05, ** *p* < 0.01. Data shows mean ± SEM, n ≥ 3.

As detailed above, HSC were first evident in fetal BM from E18.5, albeit at an extremely low frequency, with their frequency and number significantly increasing following birth (Figure 2a). Similar to the enlarged progenitor pool in OPN^-/-^ fetal BM, the proportion of HSC was also significantly increased at D2 and D4 in the absence of OPN (Figure 3a). As OPN^-/-^ neonatal mice were smaller and had a significantly lower BM cellularity (Figure S2), this resulted in an equivalent HSC pool to WT BM. Similar to adult BM, and in contrast to fetal E17.5 BM, cell cycle analysis of D2 BM revealed that the absence of OPN resulted in significantly less HSC in G0 and more in G1; suggesting OPN inhibits HSC proliferation in situ (Figure 3b). Notably, there was no difference in the ability of transplanted WT fetal liver HSC to home to the BM of WT or D2 pups devoid of OPN (Figure S1f), demonstrating the increased neonatal HSC pool in OPN^-/-^ mice is not due to an altered ability of HSC to migrate to OPN^-/-^ BM. Collectively, the data suggests HSC in OPN^-/-^ mice cycle faster to produce a higher proportion of HSC in neonatal BM.

### 3.4. Endogenous Activation of α_9_β_1_ is Up-Regulated on Fetal BM Stem and Progenitors

**Figure 4:**
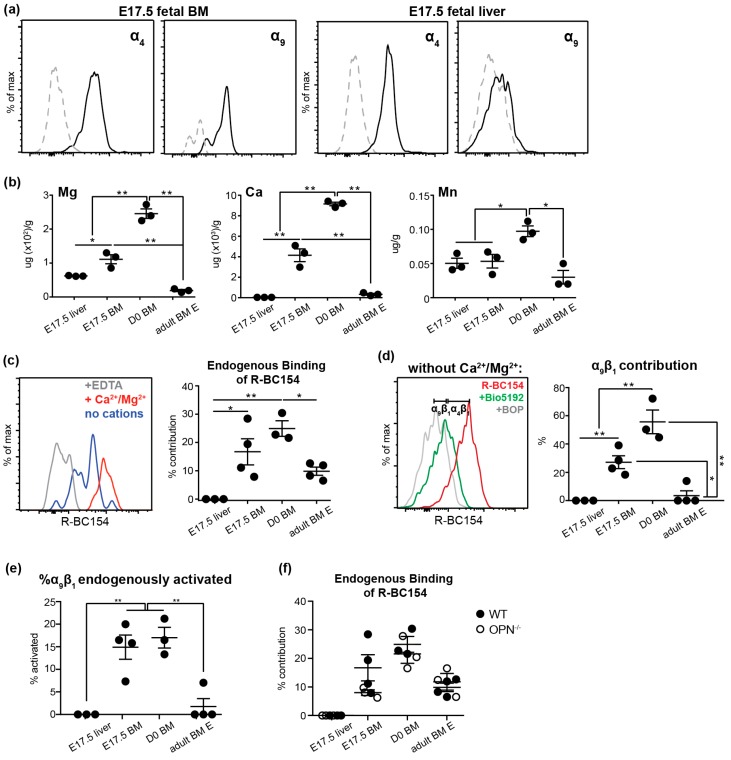
α_4_β_1_ and α_9_β_1_ are highly expressed and endogenously activated on fetal BM stem and progenitor cells. (**a**) Representative histograms of α_4_ and α_9_ (black lines; grey lines, appropriate isotype controls) expression on E17.5 fetal BM and liver LSK cells. (**b**) Quantitative analysis of magnesium, calcium and manganese in fetal liver, fetal BM and adult BM by coupled plasma mass spectrometry (ICPMS). (**c**) A representative histogram of R-BC154 binding to E17.5 fetal BM LSK cells with (red line, maximum binding) or without (blue line, endogenous activation) 1mM Ca^2+^/Mg^2+^, or in the presence of 10mM EDTA to provide the base line (grey) and the endogenous R-BC154 binding during ontogeny was plotted. (**d**) A representative histogram demonstrating the endogenous activation of α_9_β_1_ and α_4_β_1_ on 17.5 fetal BM LSK cells (red line, combination of endogenous α_9_β_1_ and α_4_β_1_ activation; difference between grey and green line, α_9_β_1_ activation and difference between green and red line, α_4_β_1_ activation) and endogenous α_9_β_1_ contribution quantified throughout ontogeny. (**e**) The proportion of endogenously activated α_9_β_1_ of total α_9_β_1_ on 17.5 fetal BM LSK cells. (**f**) Endogenous integrin activation on WT (closed black circles) and OPN^-/-^ (open circles) stem and progenitors during ontogeny. * *p* < 0.05, ** *p* < 0.01. Data shows mean ± SEM, n ≥ 3.

To identify the potential mechanism of trOPN-mediated fetal HSC migration and maintenance, we investigated the expression and activity of two of its known receptors; α_4_ and α_9_ integrins. Expression of both α_4_ and α_9_ were confirmed by flow cytometry on fetal BM and liver stem and progenitors at E17.5 (Figure 4a). However, while the expression of α_4_ was high on stem and progenitors in both fetal BM and liver, α_9_ was much lower in liver suggesting a more prominent role of the trOPN/α_9_ axis in developing BM. Integrins exist in three conformational states: Inactive with low affinity, active or primed with high affinity, and ligand-occupied [36]. Thus, despite α_4_ and α_9_ being expressed on fetal stem and progenitors, it is important to assess their activation state. We previously synthesized a fluorescent integrin antagonist, R-BC154, which specifically binds only to activated α_4_β_1_ and α_9_β_1_; this occurs due to the presence of divalent metal cations [37]. Thus, R-BC154 can be used as an activation-dependent probe for α_4_β_1_ and α_9_β_1_. In adult BM, a high concentration of divalent metal cations is maintained in the endosteal region in situ, resulting in R-BC154 binding to endogenously activated α_4_β_1_ and α_9_β_1_ on HSC and HSC progenitors [32]. Similarly, calcium, magnesium and manganese were also present in fetal BM and concentrations were significantly higher compared to both fetal liver as well as adult BM (Figure 4b). These results suggest integrin activation may be differentially regulated on fetal BM HSC by different microenvironments, potentially contributing to their embryonic migration. Indeed, staining with R-BC154 in the absence of exogenous cations demonstrated α_4_β_1_ and α_9_β_1_ were endogenously activated on WT fetal BM stem and progenitors but not their liver counterparts (Figure 4c). Notably, the level of endogenous activation on BM was increased at birth to levels significantly higher than adult BM (Figure 4b) and may be attributed to the greater concentration of activating divalent metal cations in D0 BM (Figure 4b). To distinguish between endogenous activation of α_4_β_1_ and α_9_β_1_, cells were treated with R-BC154 in combination with an excess of the selective α_4_β_1_ inhibitor Bio5192 [38]. As Bio5192 is significantly more potent than R-BC154, all α_4_β_1_ integrin sites are blocked by Bio5192 and any remaining R-BC154 signal observed can be attributed to α_9_β_1_ [32]. Over 20% and 50% of total R-BC154 binding occurred through endogenously activated α_9_β_1_ on WT fetal and neonatal BM stem and progenitors, respectively, while less than 2% occurred through α_9_β_1_ on their adult counterparts (Figure 4d). Furthermore, a significantly higher proportion of total α_9_β_1_ expressed by WT fetal and neonatal BM stem and progenitor cells was endogenously activated (>15%) compared to their adult counterparts (<2%; Figure 4e). Collectively, the data suggests the trOPN/ α_9_β_1_ interaction on HSC is more pronounced during early development with the role of α_4_β_1_ becoming more prevalent in adulthood. 

Since α_4_ and α_9_ integrins can also bind to other ligands such as VCAM-1 [39,40], we assessed whether the absence of OPN resulted in altered endogenous α_4_ and α_9_ activity, which could lead to dysregulation of stem and progenitor cells in OPN^-/-^ mice through indirect means. However, no difference in endogenous integrin activation was detected between WT and OPN^-/-^ stem and progenitor cells during ontogeny (Figure 4f), suggesting the increased stem and progenitor pool observed in OPN^-/-^ fetal, neonatal and adult BM is not due to dysregulated endogenous α_4_β_1_ and α_9_β_1_ activation. Collectively, these results suggest the activation of both α_4_β_1_ and α_9_β_1_ is up-regulated on fetal stem and progenitors in BM, which then permits binding to trOPN and maintenance of the HSC pool in developing BM.

## 4. Discussion

Similar to adult HSC, fetal HSC are heavily influenced by their microenvironments [25], with each hematopoietic organ educating HSC and modifying hematopoiesis during ontogeny. Fetal liver HSC are a unique stem cell population with more than 40% of stem cells being in the cell cycle, compared to <3% of adult HSC [41,42]. Most recently, the RNA-seq profile of fetal liver HSC was compared to adult BM, and consistent with the greater proportion of cycling cells, genes highly expressed in fetal liver were enriched for biological processes related to DNA replication and cell proliferation [43]. Therefore, fetal liver needs to provide a conducive microenvironment for HSC expansion. For example, compared with the adult BM microenvironment, insulin-like factor 2 and angiopoietin-like factors in fetal liver provide a unique set of stimulating signals to HSC [44,45]. In accordance with these tissue-specific roles, our current study revealed minimal levels of trOPN in fetal liver. Furthermore, the binding partners of trOPN, α_4_β_1_ and α_9_β_1_, were not endogenously activated on fetal liver HSC due to locally low divalent metal cation concentrations. This data is in contrast with previously reported high expression of OPN by cultured fetal liver cells, which could be due to the in vitro culture conditions [23]. As a consequence, the absence of OPN does not affect the size of the progenitor and HSC pools or lineage commitment in fetal liver. In contrast, OPN plays a pivotal role in regulating fetal BM HSC and progenitors in vivo.

Murine fetal skeletal development begins at E12.5, when mesenchymal cells first give rise to chondrocytes that create a cartilaginous framework for the skeleton [46]. These structures are occupied by differentiated myeloid cells and represent a transient site for hematopoietic development [47]. Later, through endochondral ossification, the mineralized cartilage is replaced by osteoblasts generating calcified bone, and vascularization is established in the developing bones. More and more HSC are attracted to these developing BM cavities, completing the transition of hematopoiesis from the fetal liver [48]. Eventually, the BM becomes the permanent and predominant location for hematopoiesis throughout adulthood. Our data revealed OPN, and specifically trOPN, is highly expressed in fetal BM at trabecular bone surfaces. 

Previous studies show OPN^-/-^ embryos were smaller than wild type at all gestational ages [49]. We show that this is retained post-birth, when OPN^-/-^ neonatal pups weighed significantly less than their wildtype counterparts and had significantly lower BM cellularity after D2. Similar to that previously described in the adult [5,13], an enlarged progenitor pool was demonstrated in murine E17.5 BM in the absence of OPN, but with an accompanying decrease in the content of CD45^+^ hematopoietic cells and Gr1^+^ granulocytes. This is unlikely due to altered colonization of hematopoietic cells to BM, as no difference was observed at E16.5. A more likely explanation is impaired progenitor differentiation in the OPN null BM microenvironment. This finding is similar to that observed in mice lacking α_9_, which also have a granulocyte differentiation defect in fetal BM [50], with phosphorylated STAT3, a potential downstream intermediate being significantly reduced in the absence of α_9_ [50], but stimulated by OPN overexpression [51,52]. A similar role for OPN in lineage commitment has been previously reported in both adult [5] and aged [53] mice. Furthermore, OPN plays a functional role in HSC proliferation throughout life, with its absence driving HSC from G0 to G1 in both young [13,54] and aged [54] mice; with the current study extending this to newborns. Together, this supports a central regulative mechanism for OPN that is preserved at different developmental stages to balance maintenance of the HSC pool with blood cell production. 

The ability of OPN to regulate cells is dependent on its ability to interact with binding partners including α_4_β_1_ and α_9_β_1_ [17]. Binding to integrins is completely dependent on their activation state [32,55]. Whilst α_4_β_1_ and α_9_β_1_ are expressed by a variety of cell types, including HSC and progenitors, they are predominantly in an inactive state [32]. The current study demonstrates that compared to the adult, a significantly higher proportion of α_9_β_1_ integrin expressed on fetal and newborn stem and progenitors is endogenously activated, suggesting an important role of α_9_β_1_ in fetal hematopoiesis. 

Osteopontin is pivotal in vasculogenesis [19,27], which may indirectly control hematopoiesis during ontogeny. Analysis of bone formation in OPN^-/-^ mice revealed no differences between OPN^-/-^ and wild type pups in the content of osteoblast lineage cells (Figure S3a,b), femoral length (Figure S3c) or bone marrow cavity development at the primary ossification centre (Figure S3d). Similarly, the absence of OPN did not have an impact on fetal BM vascularization or endothelial marker expression (Figure S3e–h). Furthermore, although endogenous OPN stimulates cultured tumor cells to produce more cytokines in vitro, such as SDF-1α and VEGF, the absence of OPN did not affect their expression in fetal BM in vivo (Figure S3i). In OPN knockout mice, other bone proteins, such as bone sialoprotein (BSP) or osteoadherin, which bind to α_v_β_3_ integrin and have a role in bone formation [56,57] may compensate for OPN. Furthermore, besides trOPN, vascular cell adhesion protein-1 (VCAM-1) is a ligand for α_4_β_1_ and α_9_β_1_ and involved in HSC mobilization [39,40]. We demonstrated VCAM-1 is expressed in OPN^-/-^ fetal BM, and may bind to α_4_β_1_ and α_9_β_1_ and thereby mediate HSC trafficking (Figure S3c). The benefit of such network-based regulation is to avoid the huge impact of a single gene mutation on HSC, as compensation using alternate mechanisms maintains normal hematopoiesis. Furthermore, a network-based regulation ensures HSC are able to respond to an urgent need for blood production and at the same time take advantage of a feedback system to prevent excessive stimulation resulting in progression to diseases such as cancer. 

In summary, the current study demonstrated trOPN in fetal BM and endogenous activation of its receptors α_4_β_1_ and α_9_β_1_ integrins on fetal BM HSC and progenitors. Similar to the adult, OPN is an important regulative component in fetal BM HSC and progenitor pools and is involved in progenitor differentiation and maintenance of HSC quiescence.

## Supplementary Materials

The following are available online at https://www.mdpi.com/2073-4409/8/9/985/s1, Figure S1: The incidence and content of HSC and progenitors in fetal liver; Figure S2: Mouse weight and BM cellularity in OPN^-/-^ mice; Figure S3: The absence of OPN does not change the fetal BM HSC residential microenvironment.
