# Supplementary material for: Osteopontin is An Important Regulative Component of the Fetal Bone Marrow Hematopoietic Stem Cell Niche
Source: Cells. 2019 Aug 27;8(9):985. doi: 10.3390/cells8090985 (PMC6770910; doi:10.3390/cells8090985)
Supplement: Supplementary file 1 [file cells-08-00985-s001.pdf]

Supplemental Materials

Methods

Mice

OPN$^{-/-}$ cdh5 td-tomato and cdh5 td-tomato wildtype control mice were bred on a C57BL/6J (C57, Ly5.2) background at Monash Animal Research Platform (Monash University, Australia). All experiments were approved by the Monash Animal Research Platform ethics committee. Embryos were harvested at E17.5 with timed pregnancies set up as previously described [1]. Specifically, (OPN$^{-/-}$) cdh5-wt td-tomato males were mated with (OPN$^{-/-}$) cdh5-cre td-tomato females for the generation of experimental fetuses. Pregnancy was confirmed at E12.5 and pregnant mice were injected intraperitoneally with Tamoxifen (Sigma, T5648) at a dose of 80mg/kg (10% EtOH in soybean oil) at E12.5 and E14.5. After harvest, embryos were genotyped for the presence of cre in individual pups.

Micro-computed tomography imaging of fetal and neonatal BM

The cationic contrast agent CA4+ salt was synthesized as previously described [2, 3]. A working solution of CA4$^+$ was prepared by dissolving 15 mg of the CA4$^+$ trifluoroacetate salt (8.29 μmol) in 400 μl de-ionized water, followed by acidification to ~pH 1 with 1-2 μl of 3 M HCl. The sample was lyophilized and the HCl salt was resuspended in 500 μl de-ionized water and treated with 1 M sodium acetate solution (33.2 μl, 33.2 μmol). The pH was adjusted carefully with 3 M NaOH to pH 7, the osmolarity was balanced to ~400 mOsm/kg with NaCl solution and then diluted to a final concentration of 12 mg/ml with de-ionized water. Fixed fetal and neonatal femurs were submerged in CA4+ solution overnight at 4 °C. The femurs were removed from the CA4+ solution and imaged using SkyScan 1076 system (Bruker-micro-CT, Kontich, Belgium). Images were acquired using the following settings: 9 μm voxel resolution, 0.5 mm aluminum filter, 48 kV voltage and 200 μA current, 2600 ms exposure time, rotation 0.7°, and frame averaging = 1. Images were reconstructed and analyzed using SkyScan software programs NRecon (version 1.6.8.0), DataViewer (version 1.4.4), and CT Analyser (version 1.11.8.0). Scanning images were reconstructed using NRecon with the threshold of 0-0.025. After reconstruction, regions of interest were defined using CTAn Analyser. Bone marrow cavity volume and total femoral tissue volume was measured in a region with length 70% of total femoral length, centred to avoid the proximal and distal epiphyses. Since bone tissue density increases during development, the minimal threshold used to define bone was increased based on the developmental age: 160 for D0, 180 for D2, 200 for D4 and 220 for D6 and D8 samples. The same parameters were applied to both WT and KO samples.

Osteoblastic lineage analysis on E17.5 fetal BM

Fetal BM osteoblasts were harvested and analyzed as previously described [4] with minor modifications. Specifically, isolated fetal bones were cut into small pieces and treated sequentially with 4mM EDTA (at 37 °C for 10 minutes) for 3 rounds and collagenase (3mg/ml at 37 °C for 10 minutes) for 2 rounds, with PBS washes between each round. Bone fragments were treated with collagenase (3 mg/ml at 37 °C for 15 minutes) and the cell suspension was isolated for osteoblastic lineage analysis. Cells were stained for lineage markers (anti-CD45, CD31, TER119), anti-Sca-1-PECy7 (0.3μg/ml; Biolegend) and anti-CD166-FITC (2μg/ml; eBioscience), washed, resuspended in PBS 2% Serum containing propidium iodide (PI; 0.05μg/ml; Life Technologies) and analyzed by flow cytometry.

BM vascularization

E17.5 fetal BM sections from $OPN^{-/-}$ cdh5 td-tomato and cdh5 td-tomato wildtype controls were imaged using a confocal microscope (Nikon). Image analysis of $tomato^{+}$ vasculature was performed using Fiji software.

Expression of endothelial cell markers on fetal endothelial cells

For analysis of endothelial cell surface markers, E17.5 fetal BM cells from $OPN^{-/-}$ cdh5 td-tomato and cdh5 td-tomato wildtype controls were initially gated for $CD45^{-}B220^{-}tomato^{+}$ endothelial cells and then analyzed for CD31 (anti-CD31 BV510 1µg/ml; BD), Endomucin (anti-Endomucin eFluor660 5µg/ml; eBioscience) and VEGFR2 (anti-VEGFR2 BV421 10µg/ml; R&D) expression by flow cytometry.

Quantification of SDF-1α, VEGF and VCAM-1

E17.5 fetal BM supernatants from $OPN^{-/-}$ and wildtype controls were assessed for SDF-1α, VEGF and VCAM-1 expression using ELISA kits purchased from R&D and performed according to the manufacturer's instructions. Each biological repeat was run in duplicate for each analysis.

Figures

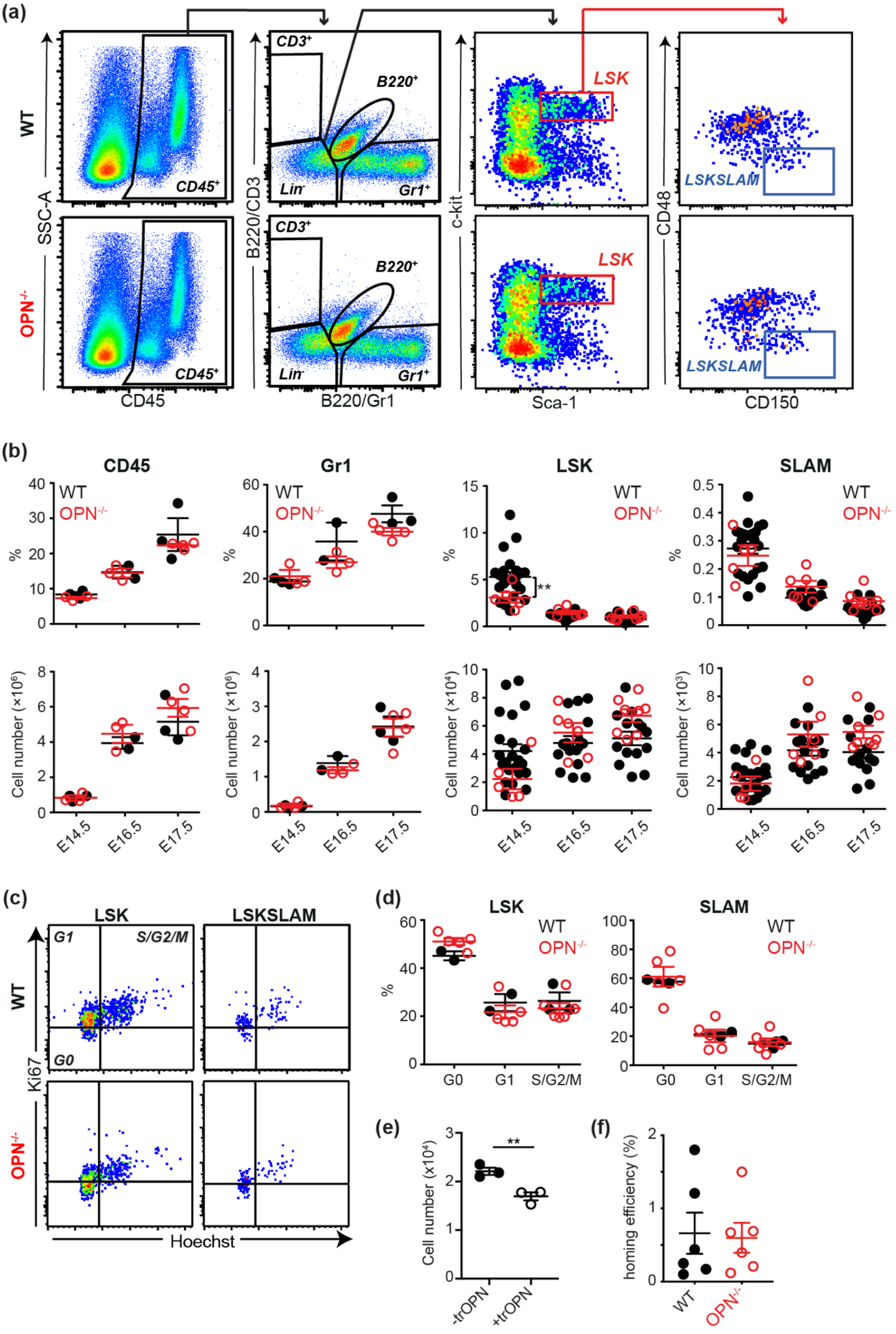

(a)
WT
OPN-/-
SSC-A
CD45
CD45+
B220/CD3
B220/Gr1
CD3+
B220+
Lin-
Gr1+
c-kit
Sca-1
LSK
CD48
CD150
LSKSLAM
(b)
CD45
Gr1
LSK
SLAM
%
Cell number (×10^6)
Cell number (×10^4)
Cell number (×10^3)
E14.5
E16.5
E17.5
**
(c)
LSK
LSKSLAM
G1
S/G2/M
G0
Ki67
Hoechst
(d)
LSK
SLAM
G0
G1
S/G2/M
(e)
Cell number (x10^4)
-trOPN
+trOPN
**
(f)
homing efficiency (%)
WT
OPN-/-

Figure S1. The incidence and content of HSC and progenitors in fetal liver. (a) Representative flow cytometric analysis of $CD45^+$ hematopoietic cells, mature blood cells, progenitors (LSK cells, red) and HSC (LSKSLAM cells, blue) and (b) their incidence and content in the liver. (c) Representative cell cycle analysis with ki67 and Hoechst on E17.5 liver LSK and LSKSLAM cells and (d) quantified data. Each dot represents the average value for a litter. WT: closed black circle; $OPN^{-/-}$: open red circle. (e) Total cell number after 6 days culture of E14.5 WT fetal liver LSKSLAM cells with and without trOPN. (f) Homing of WT E14.5 fetal liver LSKSLAM into BM of D2 WT or $OPN^{-/-}$ pups. Data shows mean±SEM, n≥3.

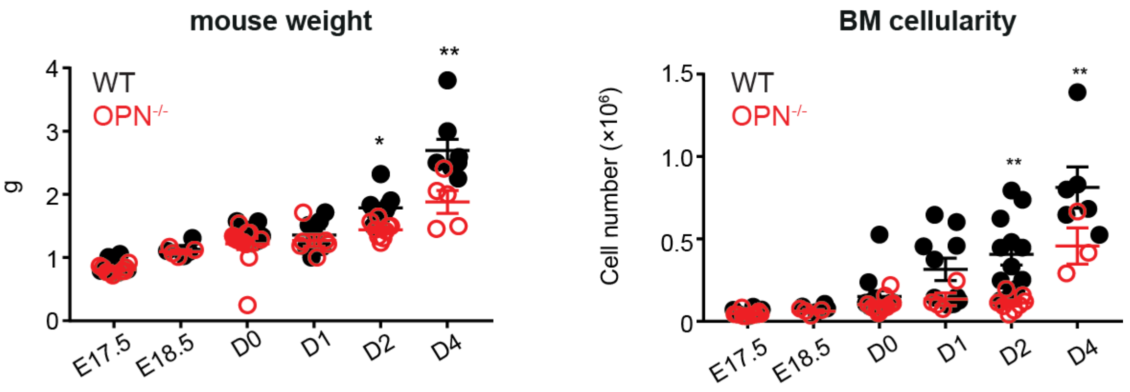

Figure S2. Mouse weight and BM cellularity of OPN$^{-/-}$ mice. BM cellularity is normalized using mouse weights. Each dot represents the average value for a litter (for fetal) or an individual mouse from different litters (for newborn). WT: closed black circle; OPN$^{-/-}$: open red circle; *$p<0.05$, **$p<0.01$. Data shows mean±SEM, n≥3.

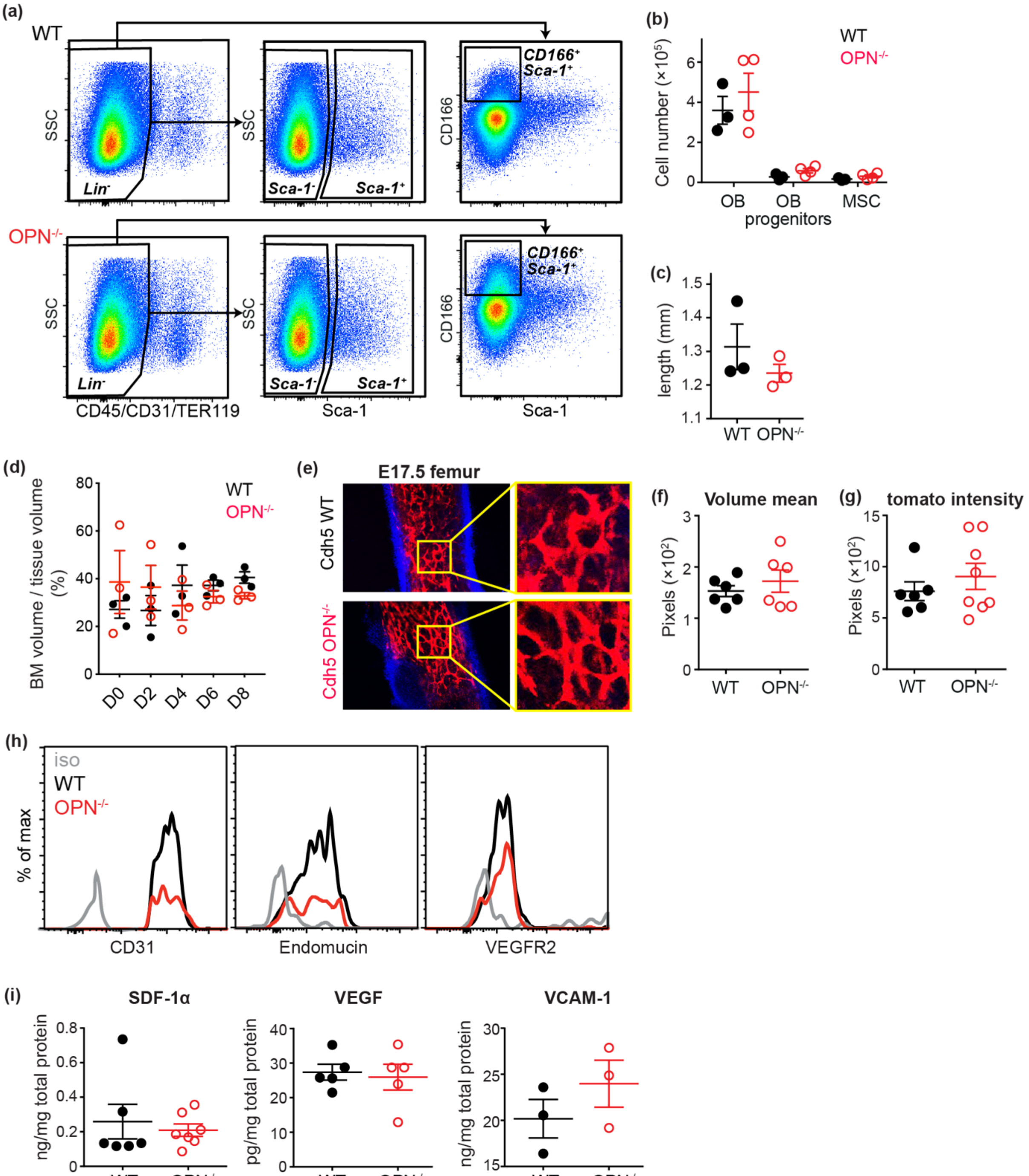

Figure S3. The absence of OPN does not change the fetal BM HSC microenvironment. (a) A representative flow cytometric analysis of osteoblastic lineages (OB, CD45$^-$CD31$^-$TER119$^-$Sca-1$^-$), OB progenitors (CD45$^-$CD31$^-$TER119$^-$Sca-1$^-$CD166$^+$), mesenchymal stem cells (MSC, CD45$^-$CD31$^-$TER119$^-$Sca-1$^+$) and (b) their content in E17.5 fetal BM of WT and OPN$^{-/-}$ mice. (c) Femoral length, including cartilaginous epiphyses, of E17.5 wildtype and OPN$^{-/-}$ pups. (d) Bone marrow cavity volume as a proportion of femoral primary ossification centre volume as determined by micro-CT. (e) Representative 3D confocal image of E17.5 BM from cdh5-td-tomato and cdh5-td-tomato/OPN$^{-/-}$ mice depicting tomato$^+$ vasculature (red). Quantitation of (f) volume mean and (g) tomato intensity between OPN$^{-/-}$ cdh5 td-tomato and cdh5 td-tomato wildtype control mice was performed with Image J. (h) Representative histogram analysis of CD31, Endomucin and VEGFR2 expression on tomato$^+$CD45$^-$B220$^-$

endothelial cells. (c) SDF-1α, VEGF and VCAM-1 quantitation in E17.5 BM supernatant. WT: closed black circle; OPN$^{-/-}$: open red circle. Data is the mean±SEM, n≥3.

References

1. Cao, H.; S. Y. Heazlewood; B. Williams; D. Cardozo; J. Nigro; A. Oteiza, and S. K. Nilsson. The Role of Cd44 in Fetal and Adult Hematopoietic Stem Cell Regulation. *Haematologica* **2016** *101*, 26-37.
2. Stewart, R. C.; A. N. Patwa; H. Lusic; J. D. Freedman; M. Wathier; B. D. Snyder; A. Guermazi, and M. W. Grinstaff. Synthesis and Preclinical Characterization of a Cationic Iodinated Imaging Contrast Agent (Ca4+) and Its Use for Quantitative Computed Tomography of Ex Vivo Human Hip Cartilage. *J Med Chem* **2017** *60*, 5543-55.
3. Joshi, N. S.; P. N. Bansal; R. C. Stewart; B. D. Snyder, and M. W. Grinstaff. Effect of Contrast Agent Charge on Visualization of Articular Cartilage Using Computed Tomography: Exploiting Electrostatic Interactions for Improved Sensitivity. *J Am Chem Soc* **2009** *131*, 13234-5.
4. Nakamura, Y.; F. Arai; H. Iwasaki; K. Hosokawa; I. Kobayashi; Y. Gomei; Y. Matsumoto; H. Yoshihara, and T. Suda. Isolation and Characterization of Endosteal Niche Cell Populations That Regulate Hematopoietic Stem Cells. *Blood* **2010** *116*, 1422-32.
